# Management of Postnatal Depression: A Systematic Review of Clinical Practice Guidelines

**DOI:** 10.1192/bjo.2025.10123

**Published:** 2025-06-20

**Authors:** Aliya Durrani, Nishani Fonseka, Ram Bajpai, Tom Kingstone, Saeed Farooq

**Affiliations:** Keele University, Staffordshire, United Kingdom

## Abstract

**Aims:** Postnatal Depression (PND) is the most prevalent mental health disorder during the postpartum period. Evidence suggests that clinical practice guidelines (CPGs) have the potential to improve the mental well-being of these women. A systematic review of the CPGs for PND, addressing both pharmacological and non-pharmacological recommendations, is currently lacking in the literature. We aim to identify the existing CPGs for the management of PND and to collect the specific recommendations reported by them.

**Methods:** We conducted this review following the guidance of the Preferred Reporting Items for Systematic Reviews and Meta-Analyses (PRISMA). A comprehensive search was performed in 5 electronic databases (Medline, Embase, PsycINFO, TRIP, and Epistemonikos) and guideline-specific websites (GIN, NICE, SIGN, and WHO) to identify the currently available English language CPGs for the management of PND, published between 2012 and 2023. General characteristics of the CPGs, as well as reported pharmacological and non-pharmacological recommendations, were extracted. The AGREE-II instrument was used to assess the methodological quality of CPGs based on a cut-off point of 70% and above.

**Results:** The search strategy identified 1096 records of which 71 were assessed for full text. We identified 19 CPGs: with only one from a lower-middle-income country (Lebanon). All CPGs recommended cognitive-behavioural Therapy (CBT) as the preferred psychological therapy based on level 1 evidence (Systematic review and meta-analysis). Pharmacological interventions were included by 17 CPGs with Selective Serotonin Reuptake Inhibitors (SSRIs) being the most recommended medication based on level 2 evidence (Randomized control trial). Nine CPGs used the “GRADE Criteria” for the determination of the strength of the recommendations. Only three CPGs incorporated Patient and Public Involvement and Engagement in the form of an advisory group. Six CPGs matched the criteria of adequate quality by achieving an overall score of ≥70%.

**Conclusion:** This review highlights the lack of evidence-based CPGs in lower-middle-income countries (LMICs), which have the largest burden of disease. The application of CPGs from higher-income countries in LMICs is challenging due to significant cultural differences and the availability of evidence from their own settings. CBT and SSRIs were the most common pharmacological and non-pharmacological interventions reported in these CPGs.

